# Solvent-Exchange Triggered Solidification of Peptide/POM Coacervates for Enhancing the On-Site Underwater Adhesion

**DOI:** 10.3390/molecules29030681

**Published:** 2024-02-01

**Authors:** Fangyan Ji, Yiwen Li, He Zhao, Xinyan Wang, Wen Li

**Affiliations:** State Key Laboratory of Supramolecular Structure and Materials, College of Chemistry, Jilin University, Changchun 130012, China; jify22@mails.jlu.edu.cn (F.J.); liyiwen122723@163.com (Y.L.); hezhao23@mails.jlu.edu.cn (H.Z.); xinyan22@mails.jlu.edu.cn (X.W.)

**Keywords:** peptide, polyoxometalate, coacervate, solvent exchange, underwater adhesive

## Abstract

Peptide-based biomimetic underwater adhesives are emerging candidates for understanding the adhesion mechanism of natural proteins secreted by sessile organisms. However, there is a grand challenge in the functional recapitulation of the on-site interfacial spreading, adhesion and spontaneous solidification of native proteins in water using peptide adhesives without applied compressing pressure. Here, a solvent-exchange strategy was utilized to exert the underwater injection, on-site spreading, adhesion and sequential solidification of a series of peptide/polyoxometalate coacervates. The coacervates were first prepared in a mixed solution of water and organic solvents by rationally suppressing the non-covalent interactions. After switching to a water environment, the solvent exchange between bulk water and the organic solvent embedded in the matrix of the peptide/polyoxometalate coacervates recovered the hydrophobic effect by increasing the dielectric constant, resulting in a phase transition from soft coacervates to hard solid with enhanced bulk cohesion and thus compelling underwater adhesive performance. The key to this approach is the introduction of suitable organic solvents, which facilitate the control of the intermolecular interactions and the cross-linking density of the peptide/polyoxometalate adhesives in the course of solidification under the water line. The solvent-exchange method displays fascinating universality and compatibility with different peptide segments.

## 1. Introduction

Sessile organisms can fix themselves on various solid surfaces through secreting adhesive proteins [1,2,3], which have become inspired sources for the generation of biomimetic underwater adhesives serving as surgical glue or sealant [4,5,6,7,8,9]. Up to now, a large number of artificial underwater adhesives, such as polymers [10,11,12] recombinant proteins [13,14,15], and peptide adhesives [16,17,18,19], have been designed and synthesized. Polymer adhesives hold the advantages of reliable adhesion and easy-to-implement synthesis, but their biocompatibility and biodegradability are usually unsatisfactory [20,21]. Recombinant protein adhesives are typically biocompatible and degradable counterparts, which rarely cause apparent fibrosis, inflammation, or necrosis [22,23,24,25,26]. However, the synthesis of the recombinant proteins often involves a complicated process; long-term, low yield and poor cost-efficiency [27,28]. In this vein, short peptides are ideal candidates for filling the gap between polymer and recombinant protein adhesives owing to their inherent biocompatibility, biodegradability, structural predictability, sequence customizability, composition diversity, synthetic feasibility and adjustable bioactivity [29,30]. More importantly, short peptides hold the potential in dissecting the contribution of individual residues and assessing their synergy benefiting from the relatively simple sequences and the reduced complexity of intra- and inter-molecular interactions in contrast to the large and complicated proteins [31,32]. Unfortunately, the short peptide-based underwater adhesives remain greatly unexplored due mainly to their poor cohesion [13,14].

To address this problem, our group reported non-covalently cross-linking copolymerization between cationic short peptides and anionic polyoxometalates (POMs) in aqueous solution [33,34]. The resulting peptide/POM supramolecular copolymers at macroscopic scale showed press-sensitive adhesion under the water line. We further revealed that the multivalent ionic and hydrogen bonds between the peptides and rigid POMs played a significant role in maintaining the interconnected network structures and improving the bulk cohesion of peptide/POM underwater adhesives. Despite this advance, achieving the on-site adhesion and spontaneous solidification (without applied compressing pressure) of the peptide/POM adhesives fully implemented in water is challenging. In stark contrast, marine organisms can unhurriedly apply protein coacervates to exert the interfacial spreading, preliminary adhesion and subsequent solidification because the condensed coacervates hold the following features [35,36,37]: (1) shear-thinning viscosity, which enables them to flow easily through the narrow conduits of organisms; (2) sufficiently cohesion, which can avoid the delivered coacervates to be rapidly lost to the surrounding water and ensure their on-site deposition onto surfaces out of bulk water; (3) low interfacial tension, allowing them to maximize the interfacial spreading and wetting on solid surfaces; (4) fluidity and deformation, enabling them to form conformal contact with the substrate surface after spreading; (5) porous internal microstructures, allows the water, ions and small molecules to permeate within the matrix of the coacervates, which is particularly important for the pH, metal ions and enzyme triggered solidification. The aforementioned properties endow the unique advantage of coacervates for balancing the trade-off relationship between interfacial adhesion and bulk cohesion of protein adhesives. Building on this knowledge, we have developed a kind of peptide/POM coacervate with the aim of reproducing the on-site delivery and spontaneous solidification. However, the solidification usually requires either specific conditions [38] or complicated molecular design [39]. Therefore, a high-priority task is to apply an easy and feasible strategy for regulating the solidification of the peptide/POM coacervates in water.

**Figure 1:**
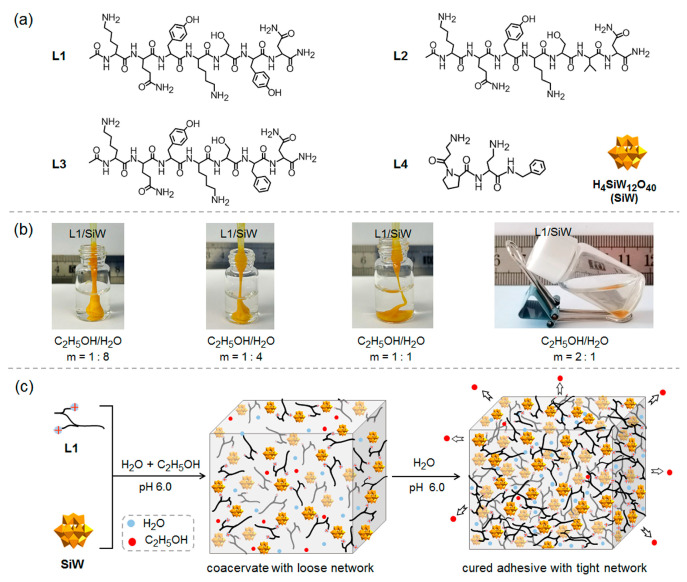
(**a**) The chemical structures of the short peptides L1, L2, L3 and L4; (**b**) The photographs of the L1/SiW complex obtained from C_2_H_5_OH-H_2_O mixed solution with different volume ratio (m) of C_2_H_5_OH to H_2_O; (**c**) The schematic drawing of the C_2_H_5_OH-H_2_O solvent exchange triggered curing of the L1/SiW coacervate.

Recently, the solvent-exchange strategy has rendered a specifically preferred solution in triggering the non-covalent interactions of polyelectrolyte coacervates or polymer solutions [40,41,42,43,44] in their internal environment and solidification. This strategy relies on the diffusion of organic solvent molecules out of the polyelectrolyte coacervates (driven by higher organic solvent concentrations in the polyelectrolyte coacervates than in the medium), holding spontaneous characteristics, universal applicability and easy operation. Here, we report several coacervates formed via non-covalent interactions between designed peptides (L1–L4, Figure 1a) and H_4_SiW_12_O_40_ (SiW, Figure 1a) in ethanol/water or DMSO/water mixed solutions with rationally optimized hydrophobic interactions. The peptide/POM coacervates could be delivered in water to establish the on-site spread and adhesion on various surfaces. Thereafter, spontaneous solidification was observed through the hydrophobic effect driven phase transition from fluid coacervate to rubber-like or a hard solid triggered by a solvent exchange between organic solvent (ethanol or DMSO) and the bulk water. The ultimate peptide/POM adhesives showed reliable underwater adhesion to various substrates (e.g., >80 kPa for stainless steel in deionized water). We also found that the adhesion strength of the peptide/POM adhesive could be triggered by controlling the hydrophobicity of individual residue of the short peptides. This work demonstrates that the non-covalent interactions and the cross-linking density of the peptide/SiW coacervates could be conveniently regulated by solvent-exchange for easy-to-implement on-site delivery, spreading, adhesion and spontaneous solidification, which are critical for improving their adhesive performance.

## 2. Results and Discussion

### 2.1. Preparation and Characterization of Peptide/SiW Coacervates

#### 2.1.1. Design of Peptide Sequences and Preparation of Peptide/SiW Coacervates

Short peptide L1 (Figure 1a), consisting of hydrophilic residues (Lys, Gln, Ser, Asn) together with two hydrophobic tyrosine (Tyr) residues, was designed in this work. Two Lys residues with strongly protonated propensity can provide cationic binding sites to electrostatically interact with the anionic SiW, giving rise to the formation of a continuously cross-linked network. The aromatic Tyr residues can strengthen the intermolecular interaction of the peptide segments and the cross-linking density of the ultimate network structures through the hydrophobic effect and the probable π-π stacking. The other residues (Gln, Ser, Asn) can also contribute additional intermolecular hydrogen bonds to enhance the structure stability. All the amino acid residues encoded in the peptide sequence have the possibility to attach to the surface of various substrates via adaptive interactions [45]. Two analogous peptides were designed by replacing the tyrosine residue near the C-termini of L1 with a relatively hydrophilic valine (Val) in L2 and a relatively hydrophobic phenylalanine (Phe) in L3, respectively, to evaluate the effect of single residue on the adhesion performance of the peptide/SiW coacervates triggered by the solvent-exchange strategy. Furthermore, one commercially available short peptide L4 was utilized to demonstrate the universality of the strategy. The purity of all the peptides was verified by High Performance Liquid Chromatograph (HPLC, Figure S1) and electrospray ionization mass spectrometry (ESI-MS, Figure S2). Before the solvent-exchange investigations, we studied the phase behavior of the co-assemblies of peptides and SiW in aqueous solution to assess their bulk cohesion. It was observed that hard or rubber-like solids were obtained after mixing the cationic peptides and the anionic SiW in an aqueous solution (pH 6.0). This result implies that the peptide/SiW complexes obtained in an aqueous solution could resist external mechanical stress and exhibit reliable bulk cohesion. We proceeded to prepare the fluid peptide/SiW coacervates by weakening the intermolecular non-covalent interactions with the aid of polar organic solvents. It is expected that the introduction of polar organic solvents into the peptide/SiW complexes can reduce the hydrophobic effect and soften the peptide/SiW complexes because most organic solvents possess lower dielectric constant (ε) relative to water. Taking L1/SiW as an example, the softening effect of the ethanol on the L1/SiW complex was observed at the volume ratio (m = 1:8) of C_2_H_5_OH/H_2_O (Figure 1b) owing to favorable interactions between L1 and ethanol. With increasing the volume ratio (m) at 2:1, the mixing solution of L1 and SiW immediately became turbidity followed by liquid–liquid separation, forming a water-immiscible, dense and fluid L1/SiW complex coacervate co-existed in equilibrium with the upper transparent supernatant (Figure 1b). A transparent solution could be obtained by further increasing the volume ratio (m) over 4:1. Similarly, fluid coacervates could be obtained by mixing L2 or L3 with SiW in C_2_H_5_OH/H_2_O solution (Figure S3), respectively. In the case of L4, DMSO/H_2_O solution was utilized to prepare the L4/SiW coacervate (Figure S3).

#### 2.1.2. Characterization of L1/SiW Coacervate

**Figure 2:**
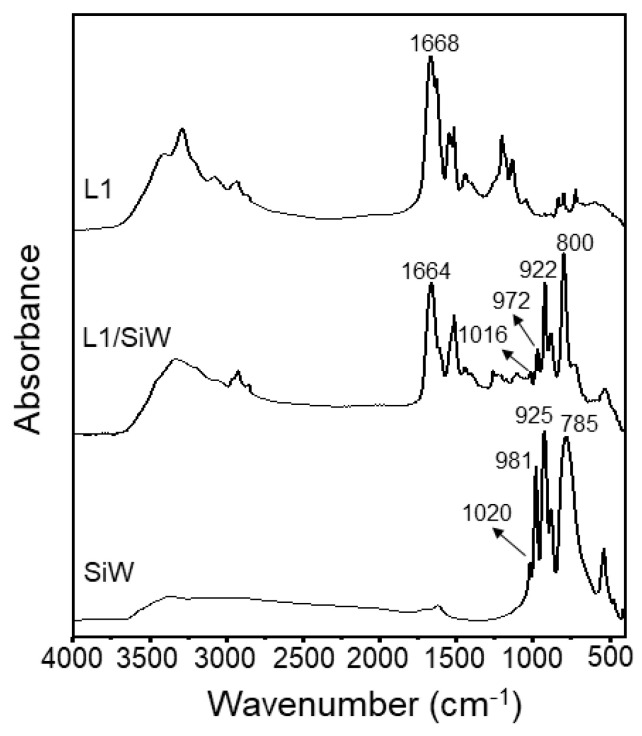
FT−IR spectra of the lyophilized L1/SiW, individual L1 and SiW.

The resulting L1/SiW coacervate was lyophilized and characterized by mass spectrometry, fourier transform infrared (FT-IR), elemental analysis (EA) and thermogravimetric analysis (TGA). The matrix-assisted laser desorption/ionization-time-of-flight mass spectrometry (MALDI-TOF-MS) of the L1/SiW sample (Figure S4) showed three peaks at 971.1, 993.1 and 1009.1, corresponding to the mass-to-charge ratio (*m*/*z*) of [M_L1_+H]^+^ fragment (calcd 718.53), [M_L1_+Na]^+^ fragment (calcd 718.53)and [M_L1_+K]^+^ fragment (calcd 718.53), respectively. This demonstrated that the peptide L1 remained intact and did not undergo any chemical reaction during the coacervation process. We further performed the negatively ionic reflector mode of electrospray ionization mass spectrometry (ESI-MS) to examine the topological integrity of SiW during the coacervation process. As illustrated in Figure S5a, L1/SiW showed the peak at *m*/*z* 718.8, corresponding to the [SiW_12_O_40_]^4−^ fragment (calcd 718.53). The MS data are almost the same as the individual SiW product (Figure S5b). This result demonstrates the structural stability of the SiW cluster during the preparation process. FT-IR spectra (Figure 2) of the lyophilized L1/SiW coacervate further demonstrated that the peptide L1 adopted a random-coil conformation within the matrix L1/SiW according to the amide I band at 1664 cm^−1^ [46] which is similar to that (1668 cm^−1^) of the individual L1 molecules. In addition, the typical vibration modes of SiW appeared at 800 cm^−1^, 922 cm^−1^, 972 cm^−1^ and 1016 cm^−1^ (Figure 2), corresponding to the ν_as_(W-O_c_-W), ν_as_(W-O_b_-W), ν_as_(Si-O_a_) and ν_as_(W=O_d_), respectively [47]. This is an indication of structural integrity of the SiW cluster. Compared with that of the SiW alone, the vibration modes of the L1/SiW in the low-frequency region showed a slight shift possibly due to the intermolecular interaction between SiW and L1 [48]. These results in combination indicate that the formation of the L1/SiW complex coacervate is driven by non-covalent interactions. The elemental analysis (EA) of the lyophilized L1/SiW powder showed C %, 19.35%; H %, 3.09%; N %, 6.08%. Thermal gravimetric analysis (TGA) showed a mass loss of 0.63% from 30 to 213.5 °C (Figure S6a), arising from the loss of crystal water embedded in the lyophilized L1/SiW powder. Taking together the EA and TGA data, the average molecular formula should be H_0.6~0.8_(H_2_C_44_H_66_N_12_O_12_)_1.6~1.7_SiW_12_O_40_·(H_2_O)_1.5~2_. The stoichiometry is almost consistent with the formation of a close to charge neutralized L1/SiW coacervate, suggesting that the ionic interactions between L1 and SiW are the dominant driving forces for the coacervation. We proposed that the presence of ethanol in the aqueous solution in our system did not lower the protonation degree of the lysine residues of L1 but significantly suppressed the hydrophobic aggregation of L1 molecules, giving rise to the formation of a less condensed structure. The primary involvement of the ethanol-mediated hydrophobic effect is further supported by the L1 analogs. As illustrated in Figure S3, peptide L2 with less hydrophobic valine can form L2/SiW coacervate in C_2_H_5_OH/H_2_O solution (m = 1:2) with a lower content of ethanol compared to L1/SiW. However, peptide L3 with more hydrophobic phenylalanine requires a much higher content of ethanol (m = 5:1) to generate L3/SiW coacervate. It is reasonable that the presence of more hydrophobic residues in the peptide sequence requires more lower ε of the mixed solution to weaken the hydrophobic effect and reduce the cross-linking density of the peptide/SiW matrix. All the coacervates were characterized by TGA (Figure S6), MS (Figure S7), FT-IR (Figure S8).

### 2.2. Solvent-Exchange Triggered Solidification of Peptide/SiW Coacervates

#### 2.2.1. Solvent-Exchange Triggered Phase Transition of L1/SiW and Its Enhanced Adhesion Performance

**Figure 3:**
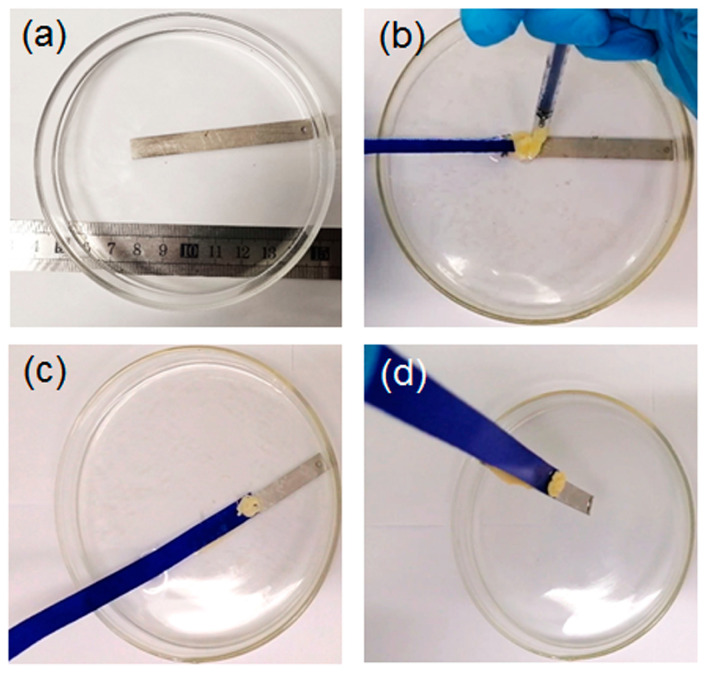
The photographs of the curing process of the L1/SiW coacervate triggered by C_2_H_5_OH-H_2_O solvent exchange. (**a**) SS plate immersed in deionized water of a glass surface dish; (**b**) injection of L4/SiW coacervate on the contact area between cloth rope and SS; (**c**) 60-min C_2_H_5_OH-H_2_O solvent exchange enables the solidification of the L1/SiW coacervate under water line; (**d**) cured L1/SiW adhesive can effectively lift the SS plate.

**Figure 4:**
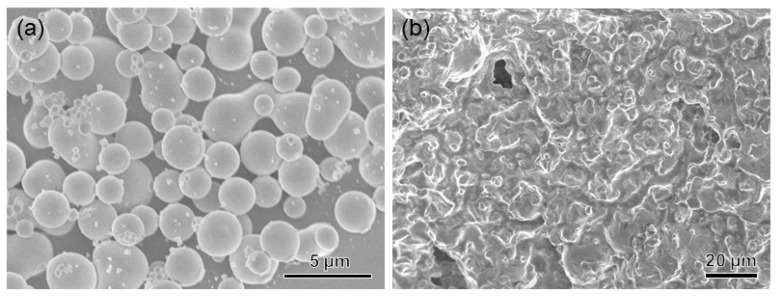
SEM images of the L1/SiW complex: (**a**) the as-prepared coacervate; (**b**) cured sample via C_2_H_5_OH-H_2_O solvent exchange.

Given the ethanol’s miscibility in water, it is expected that the as-prepared L1/SiW coacervate satisfies the solvent exchange requirements and can exert the on-site injection, interfacial spreading, contact, wet adhesion and solidification under the water line. To do this, a piece of stainless steel (SS) plate was first immersed into deionized water (pH 6.0) as shown in Figure 3a. Then a blue fabric ribbon was wet with water. The L1/SiW coacervate was extruded from a syringe and injected onto the contact area between the fabric ribbon and SS to generate a joint. One can find that the L1/SiW coacervate can precisely be deposited on the assigned position and spread on the surface very well (Figure 3b). Subsequently, the boundary was demarcated (Figure 3c) by spontaneous curing owing to the rapid solvent exchange between ethanol of the L1/SiW matrix and the bulk water on the surface of the coacervate. The cured peripheral boundary can effectively confine the internal L1 molecules and SiW clusters preventing their physical diffusion or dispersion out of the boundary during the solvent-exchange process. With increasing the setting time, the coacervate samples gradually changed into a gel-like state due to the continuous ethanol-water solvent exchange. After the L1/SiW coacervate setting for a longer time (30 min) in water, the in-situ solidification was achieved and the coacervate sample became a cured joint, which can lift the SS plate easily from water (Figure 3d). Other substrates can also be joined by the solvent-exchange strategy (Movie S1). The scanning electron microscope (SEM) experiments revealed that the microscopic morphology of the L1/SiW coacervate changed from the original condensed droplets (Figure 4a) to compact structures (Figure 4b). In the course of the ethanol–water solvent exchange, the bulk water molecules diffused into the matrix of coacervate by replacing the ethanol molecules, resulting in the increment of ε as well as the hydrophobic effect of L1 molecules. The increased hydrophobic effect contributes a remarkable energy gain and offers a critical driving force to strengthen the inter-peptide aggregation within the coacervate matrix (Figure 1c). With the continuous ethanol–water solvent exchange, the coacervate could be solidified into a hard joint with a tight structure and enhanced adhesion performance. This solvent-exchange kinetics was closely related to the adhesion strength of the L1/SiW. As shown in Figure S6, the adhesion strength of L1/SiW bonded on stainless steel (SS) is proportional to the solvent-exchange time and approaches a steady stage when the solvent-exchange time over 1 h.

**Figure 5:**
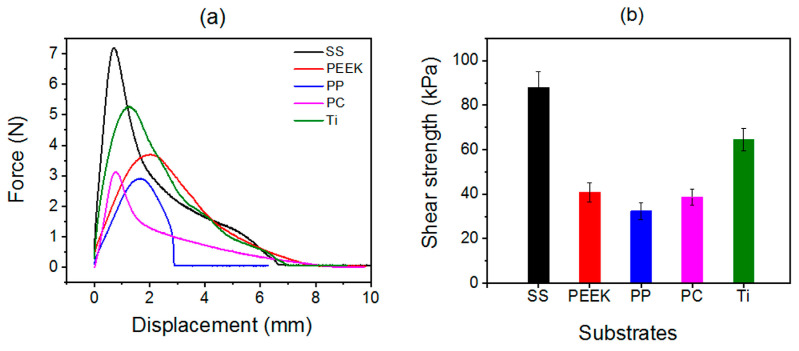
(**a**) The typical force-displacement curve for lap shear of L1/SiW bonded to different substrates at pH 6.0; (**b**) The average underwater shear adhesion strengths of the cured L1/SiW adhesive bonded to different substrates at pH 6.0.

We further utilized a material testing system to quantitatively evaluate the underwater adhesion strength of the cured L1/SiW adhesive against diverse substrates. First, the as-prepared L1/SiW coacervate samples were set between two same plates, such as stainless steel (SS), polyether–ether–ketone (PEEK), polypropylene (PP), polycarbonate (PC) and titanium (Ti) without any external compression, followed by immediately immersing into deionized water at 25 °C to cure 1 h for producing a lap shear joint. Then, the produced lap shear joint was quickly fixed on the load arm of the materials testing system equipped with a reservoir holder around the load arm to ensure all the measurement process was performed under the water line. Ultimately, the force versus displacement curves of L1/SiW can be obtained when the adhered plates were separated (Figure 5a). The measurements were performed on at least five replicates of independently prepared samples. The averaged underwater shear strength of L1/SiW was 88.0 ± 7.0 kPa for SS, 40.9 ± 4.3 kPa for PEEK, 32.4 ± 3.9 kPa for PP, 38.6 ± 3.5 kPa for PC and 56.2 ± 5.2 kPa for Ti (Figure 5b).

#### 2.2.2. Effect of Single Amino Acid Residue on Adhesion Performance of Peptide/SiW Adhesives

**Figure 6:**
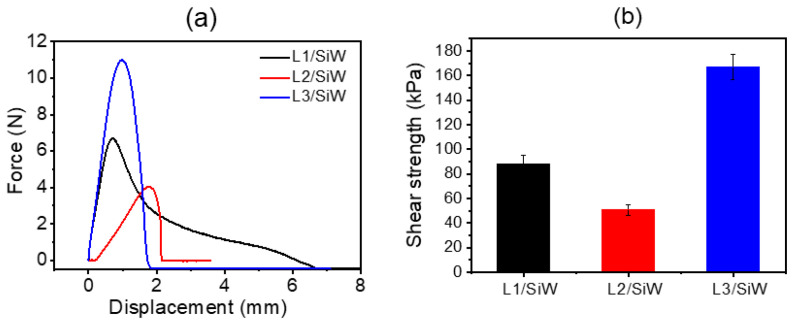
(**a**) The force-displacement curve for lap shear of different peptide/SiW bonded to SS substrates at pH 6.0; (**b**) The average underwater shear adhesion strengths of the cured peptide/SiW adhesives bonded to stainless steel substrates at pH 6.0.

Given that solvent exchange is associated with hydrophobic aggregation, we proceed to dissect the effect of single hydrophobic residue on the adhesion performance of the resulting coacervates, such as L2/SiW and L3/SiW. We scrutinized the lap shear strength of the cured L2/SiW and L3/SiW adhesives after solvent-exchange treatment. Here, SS was selected as an adherend to generate lap shear joint by setting L2/SiW and L3/SiW coacervates between two SS plates, respectively. The solvent-exchange procedures and curing time are exactly the same as that of L1/SiW. All the coacervates could be cured under the water line. We found that the underwater shear strength of the peptide/SiW adhesives showed a cumulative increment with increasing the hydrophobicity of individual residue of the short peptides from L2 across L1 to L3. As shown in Figure 6, the underwater shear strength of L2/SiW showed a modest decrease at 50.7 ± 4.3 kPa. However, a significant increase in the shear strength was observed in the case of L3/SiW (166.9 ± 10.2 kPa). It is clear that the single hydrophobic residue encoded in the peptide sequence showed an impressive effect on the adhesion performance of the peptide/SiW adhesive. Replacing a single tyrosine residue of peptide L1 with a less hydrophobic valine in L2 resulted in a decline in bulk cohesion of the cured L2/SiW. Inversely, changing one tyrosine residue into phenylalanine in L3 endowed the matrix of L3/SiW with a more hydrophobic effect. As a result, the shear strength of the cured L3/SiW is almost twofold higher than that of the L1/SiW. A plausible consequence of this difference is that the hydrophobic effect of the aromatic phenyl residue without the phenol hydroxyl group could be significantly promoted in a high-dielectric-constant solution, giving rise to enhanced bulk cohesion.

### 2.3. Universality of Solvent-Exchange Strategy

**Figure 7:**
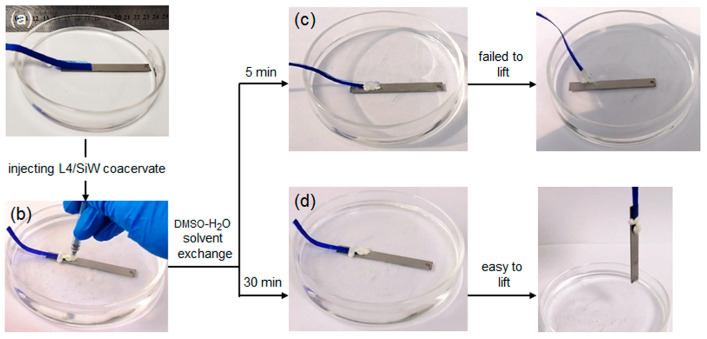
The photographs of the curing process of L4/SiW coacervate triggered by DMSO-H_2_O solvent-exchange strategy: (**a**) cloth rope and Ti plate immersed in deionized water of the glass surface dish; (**b**) underwater injection of L4/SiW coacervate on the contact area between cloth rope and Ti; (**c**) 5-min DMSO-H_2_O solvent exchange cannot produce effect joint to lift the Ti plate; (**d**) 30-min DMSO-H_2_O solvent exchange enables the effective solidification of the L4/SiW coacervate to lift the Ti plate.

Here, we take snake venom peptide L4 as an example to demonstrate the universality. As mentioned in Figure S3, L4/SiW coacervate could be prepared in a DMSO/H_2_O mixed solution. The DMSO-water solvent-exchange triggered solidification of L4/SiW coacervate was shown in Figure 7 and Movie S2. The fabric and titanium (Ti) plate were placed in a watch glass containing deionized water (Figure 7a). Then the L4/SiW coacervate was injected onto the contact area between fabric and Ti plate to form the primary spreading and contact (Figure 7b). The in-situ cured peripheral boundary can be visualized quickly owing to the interfacial solvent exchange between the DMSO of the L4/SiW matrix and the bulk water. However, the Ti plate could not be lifted within several minutes (Figure 7c) because the cured boundary reduced the solvent-exchange rate between the bulk water and the interior DMSO of the L4/SiW coacervate. By increasing the setting time over 1 h, the coacervate samples gradually cured into a hard solid with enough high cohesion. As a consequence, the Ti plate could be lifted easily (Figure 7d) from water, demonstrating the feasibility of the solvent-exchange strategy. We also identified the applicability of the solvent-exchange strategy in tap water, river water and 100 mM NaCl solution (Figure S10). This feature offers wide opportunities in that many commercially available peptides can be utilized to generate biomimetic underwater adhesives by tuning the solvophobic and solvophilic properties of peptide coacervates via changing the medium from an organic/water mixed solvent with low ε to water with relatively high ε. Actually, the physically cross-linked peptide adhesives showed strong propensity to dissociate in water, which has been explored as degradable adhesives for dura sealing and repairing [19,49].

## 3. Materials and Methods

### 3.1. Materials

Silicotungstic acid (H_4_SiW_12_O_40_, SiW) was purchased from Sinopharm Group. Short peptides, such as Ac-KQYKSYN-NH_2_ (L1), Ac-KQYKSVN-NH_2_ (L2), Ac-KQYKSFN-NH_2_ (L3), and snake venom peptide (L4), were purchased from Chengdu Yunxi Chemical Co., Ltd. All the reagents were utilized without further purification. Deionized water (18.2 Ω) was used in the experiment.

### 3.2. Preparation of the Peptide/SiW Complex Coacervates

L1/SiW: peptide L1 (97.1 mg) was dissolved in a mixed solvent of deionized water and ethanol, and the SiW (143.9 mg) was dissolved in deionized water. The solution of L1 was dropwise added into the solution of SiW by keeping the molar ratio of L1 to SiW at 2:5, the volume ratio of ethanol to water at 2:1. The pH of the mixed solution was adjusted at ~6.0 using a diluted NaOH solution. As a next step, the mixed solution was subtly heated to 50 °C for 3 min under ultrasonication followed by cooling to room temperature (25 °C). As a result, fluid L1/SiW coacervate was observed. EA of the lyophilized L1/SiW powder showed C %, 19.35%; H %, 3.09%; N %, 6.08%. TGA showed a mass loss of 0.63% from 30 to 213.5 °C. Therefore, the average molecular formula fitted with the above results should be H_0.6~0.8_(H_2_C_44_H_66_N_12_O_12_)_1.6~1.7_SiW_12_O_40_·(H_2_O)_1.5~2_.

L2/SiW: peptide L2 (90.7 mg) was dissolved in a mixed solvent of deionized water and ethanol, and SiW (143.9 mg) was dissolved in deionized water. The solution of L2 was dropwise added into the solution of SiW by keeping the molar ratio of L2 to SiW at 2:5, the volume ratio of ethanol to water at 1:2, and the final pH at 6.0 using a diluted NaOH solution. The mixed solution was subtly heated to 50 °C for 3 min under ultrasonication followed by cooling to room temperature (25 °C). As a result, fluid L2/SiW coacervate was observed at the bottom of the reaction glass bottle. EA of the lyophilized L2/SiW powder showed C %, 16.52%; H %, 2.93%; N %, 5.87%. TGA showed a mass loss of 3.98% from 30 to 198.4 °C. Therefore, the average molecular formula fitted with the above results should be H(H_2_C_40_H_66_N_12_O_12_)_1.5_SiW_12_O_40_·(H_2_O)_9~10_.

L3/SiW: peptide L3 (95.5 mg) was dissolved in a mixed solvent of deionized water and ethanol, and SiW (143.9 mg) was dissolved in deionized water. The solution of L3 was dropwise added into the solution of SiW by keeping the molar ratio of L3 to SiW at 2:5, the volume ratio of ethanol to water at 5:1, and the final pH at 6.0 using a diluted NaOH solution. The mixed solution was subtly heated to 50 °C for 3 min under ultrasonication followed by cooling to room temperature (25 °C). As a result, fluid L3/SiW coacervate was observed at the bottom of the reaction glass bottle. EA of the lyophilized L3/SiW powder showed C %, 18.76%; H %, 2.88%; N %, 6.01%. TGA showed a mass loss of 2.68% from 30 to 197.2 °C. Therefore, the average molecular formula fitted with the above results should be H_0.8_(H_2_C_44_H_66_N_12_O_12_)_1.6_SiW_12_O_40_·(H_2_O)_6~7_.

L4/SiW: peptide L4 (225.28 mg) was dissolved in 0.4 mL mixed solvent of deionized water and DMSO, and SiW (1151.27 mg) was dissolved in 0.2 mL deionized water, the solution of L4 was dropwise added into the SiW solution with charge ratio of 4:3, the volume ratio of DMSO to water at 1:2, and the final pH was controlled at 6.0 using a diluted NaOH solution. The mixed solution was subtly heated to 60 °C for 3 min under ultrasonication followed by cooling to room temperature (25 °C). As a result, fluid L4/SiW coacervate appeared at the bottom of the reaction glass bottle. EA of the lyophilized L4/SiW powder showed C %, 11.69%; H %, 1.78%; N %, 3.57%. TGA showed a mass loss of 0.87% from 30 to 77.2 °C. Therefore, the average molecular formula fitted with the above results should be H_0.2~0.4_(H_2_C_19_H_29_N_5_O_3_)_1.8~1.9_SiW_12_O_40_·H_2_O_1.5~2_.

### 3.3. Description of the Characteristics of the Peptide/SiW Complex Coacervates

FT-IR spectroscopic analysis was performed using a Bruker Optics Vertex 80V FT-IR spectrometer (Billerica, MA, USA) equipped with a DTGS detector (32 scans) at a resolution of 4 cm^−1^ by using KBr pellets. The lyophilized peptide/SiW powder samples, individual peptides and SiW powder were utilized for FT-IR measurements.

MALDI-TOF-MS data were acquired on an Autoflex speed TOF/TOF (Bruker, Billerica, MA, USA). The data were recorded in a positively ionic reflector mode.

ESI-MS was performed on the Esquire 6000 spectrometer system of Brook Dalton Company using a negative ionic reflector mode.

EA (C, H, N) was obtained on Elementar vario MICRO cube (Wallenhorst, Germany). The final average formula was calculated on the basis of three replicate results.

TGA was performed on the Q500 thermal analyzer (New Castle TA Instruments, New Castle, DE, USA) with a heating rate of 10 °C/min in the temperature range of 30–800 °C. The high-purity nitrogen was used as the carrier gas.

The morphology of the samples was studied by using a JEOL FESEM 6700F electron microscope (Tokyo, Japan) with an accelerating voltage of 15 kV. The SEM specimens were prepared by depositing the heated turbid solutions of peptide/SiW complexes on silicon slices. The resulting samples were quickly put into liquid nitrogen for 10 min and then were lyophilized under a vacuum followed by sputter-coating with platinum.

The lap shear strength was measured on the Instron 5944 material testing system with a 100 N force sensor. All lap-shearing experiments were performed at 25 °C in the aqueous medium mentioned for each test. The peptide/SiW sample was placed between two plates with compression, and the compressed plates were immediately immersed into deionized water at 25 °C for 1 h to create a lap shear joint. Subsequently, the lap shear joint was fixed on the load arm of the materials testing system equipped with a reservoir holder around the load arm. The force-displacement curves were recorded by pulling apart the joint plates up and down at a constant rate of 10 mm/min. The shear adhesion strength was calculated from the maximum detachment force at joint failure normalized by the initial contact area. The measurements were performed at least five measurements on independently prepared samples. The reported lap shear strengths are the average of five replicate measurements on independently prepared samples.

## 4. Conclusions

In summary, the solvent exchange is an effective vehicle for consecutively exerting the on-site injection, interfacial spreading, adhesion and spontaneous solidification of peptide/SiW coacervates, giving rise to compelling underwater adhesion and remarkable compatibility with various solid surfaces without applied compressive forces. We have proposed the peptide-based coacervates prepared from the mixed solution of water and polar organic solvents have loose network structures because the hydrophobic effect of the amino acid residues of the peptides could be significantly suppressed in the low-dielectric-constant solution. Upon setting the peptide/SiW coacervates in water, the solvent exchange between the organic solvents and the environmental water enhanced the dielectric constant of the interior environment of the coacervates and strengthened the hydrophobic effect and peptide aggregation, which actuated the in-situ phase transition from soft and fluid coacervates to hard or rubber-like solids with condensed structure and strong bulk cohesion. It should be noted that the adhesion strength is closely related to the kinetics of the solvent-exchange process. After curing for 60 min, the maximum underwater shear strength of the cured peptide/SiW adhesive can approach 170 kPa, which is much higher than that of the reported short peptide adhesives [50,51]. The solvent-exchange strategy is highly applicable to simplify the design of peptide-based underwater adhesives or coatings.

## Data Availability

All the data are shown in manuscript and Supplementary Materials.

## Supplementary Materials

The following supporting information can be downloaded at https://www.mdpi.com/article/10.3390/molecules29030681/s1, Figures S1 and S2: HPLC and MS data of the peptides; Figure S3: the photographs of the peptide/SiW coacervates; Figures S4 and S5: MS data of the L1/SiW coacervate; Figures S6–S8: TGA, MS and FT–IR data of the peptide/SiW coacervates; Figure S9: the time-dependent shear strength of L1/SiW; Figure S10: solvent-exchange triggered curing of L4/SiW coacervate in diverse water environment; Movies S1 and S2: the solvent-exchange triggered adhesion of L1/SiW and L4/SiW coacervates.

## Abbreviations

HPLC: high performance liquid chromatograph; ESI-MS: electrospray ionization mass spectrometry; MALDI-TOF-MS: matrix-assisted laser desorption/ionization-time-of-flight mass spectrometry; FT-IR: fourier transform infrared; EA: elemental analysis; TGA: thermogravimetric analysis; SEM: scanning electron microscope; SiW: H_4_SiW_12_O_40_; DMSO: dimethyl sulfoxide; SS: stainless steel; PEEK: polyether–ether–ketone; PP: polypropylene; PC: polycarbonate; Ti: titanium.
