# Supplementary material for: Solvent-Exchange Triggered Solidification of Peptide/POM Coacervates for Enhancing the On-Site Underwater Adhesion
Source: Molecules. 2024 Feb 1;29(3):681. doi: 10.3390/molecules29030681 (PMC10856236; doi:10.3390/molecules29030681)
Supplement: Supplementary file 1 [file molecules-29-00681-s001.zip › molecules-2755543-supplementary.pdf]

Solvent Exchange Triggered Solidification of Peptide/POM Coacervates for Enhancing the On-Site Underwater Adhesion

Fangyan Ji, Yiwen Li, He Zhao, Xinyan Wang, Wen Li*

State key lab of supramolecular structure and materials, College of Chemistry, Jilin University, Changchun 130012, China. jify22@mails.jlu.edu.cn (F. J.); 1821128082@qq.com (Y. L.); hezhao23@mails.jlu.edu.cn (H. Z.); xinyan22@mails.jlu.edu.cn (X. W.)
*Correspondence: wenli@jlu.edu.cn

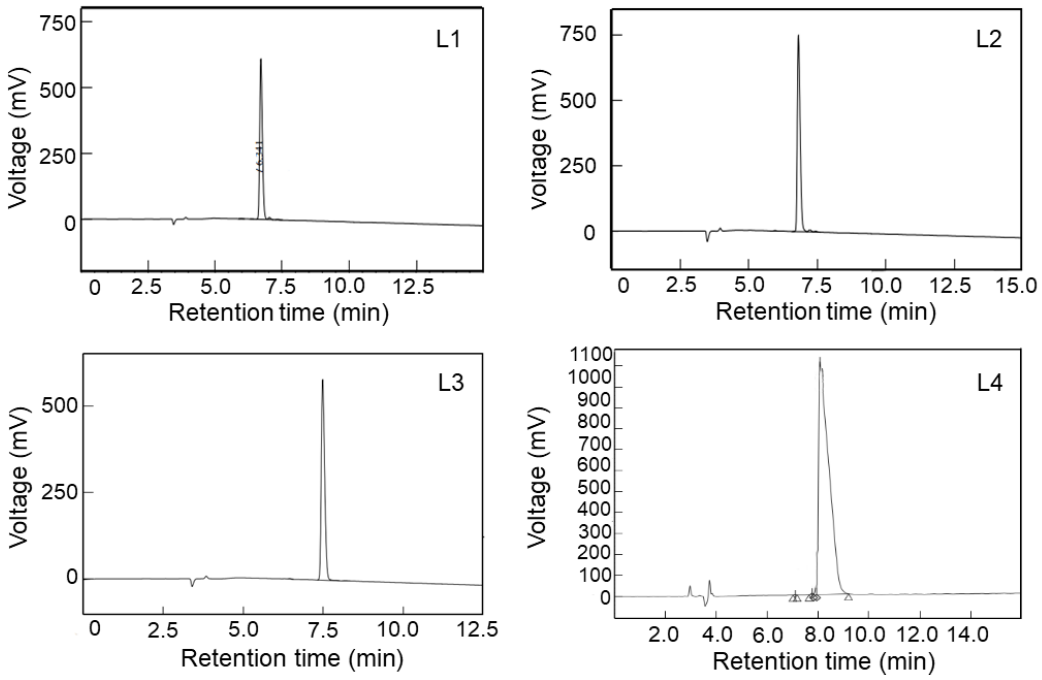

Figure S1. HPLC data of the short peptides.

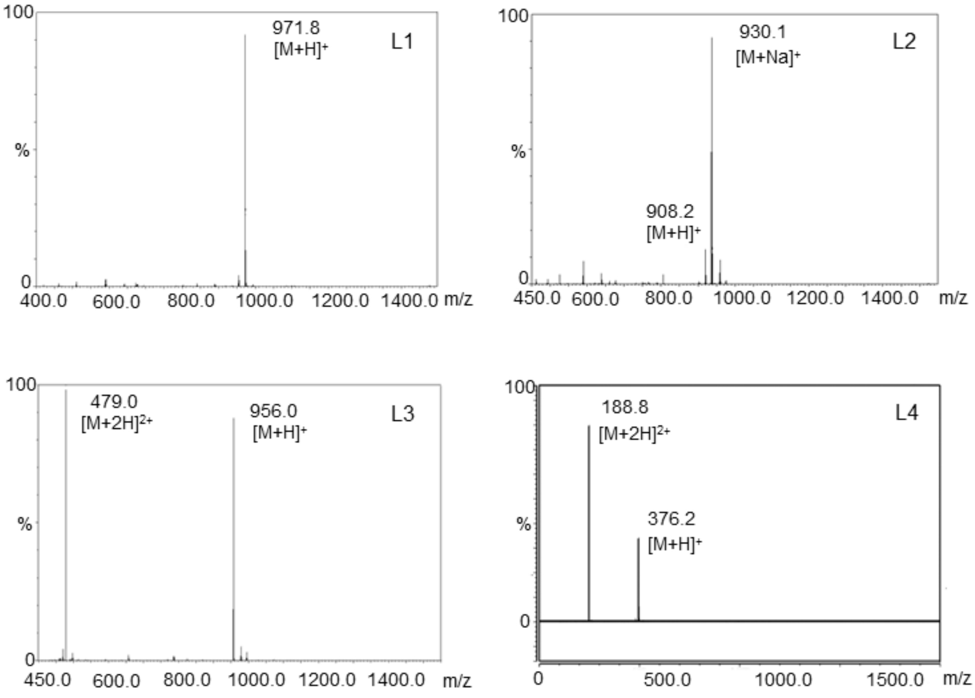

Figure S2. MS data of the short peptides.

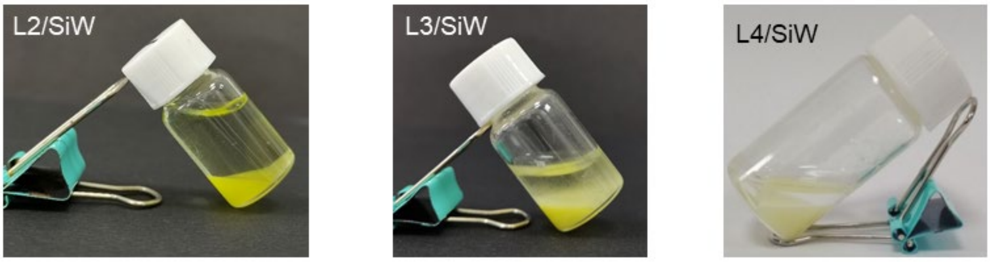

Figure S3. Photographs of L2/SiW (C_2H_5OH/H_2O 1:2), L3/SiW (C_2H_5OH/H_2O 5:1) and L4/SiW (DMSO/H_2O 1:2) coacervates.

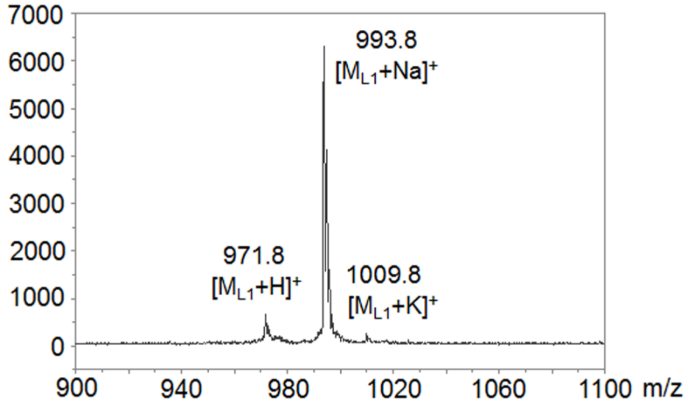

Figure S4. MALDI-TOF-MS data of L1/SiW coacervate in a positively ionic reflector mode.

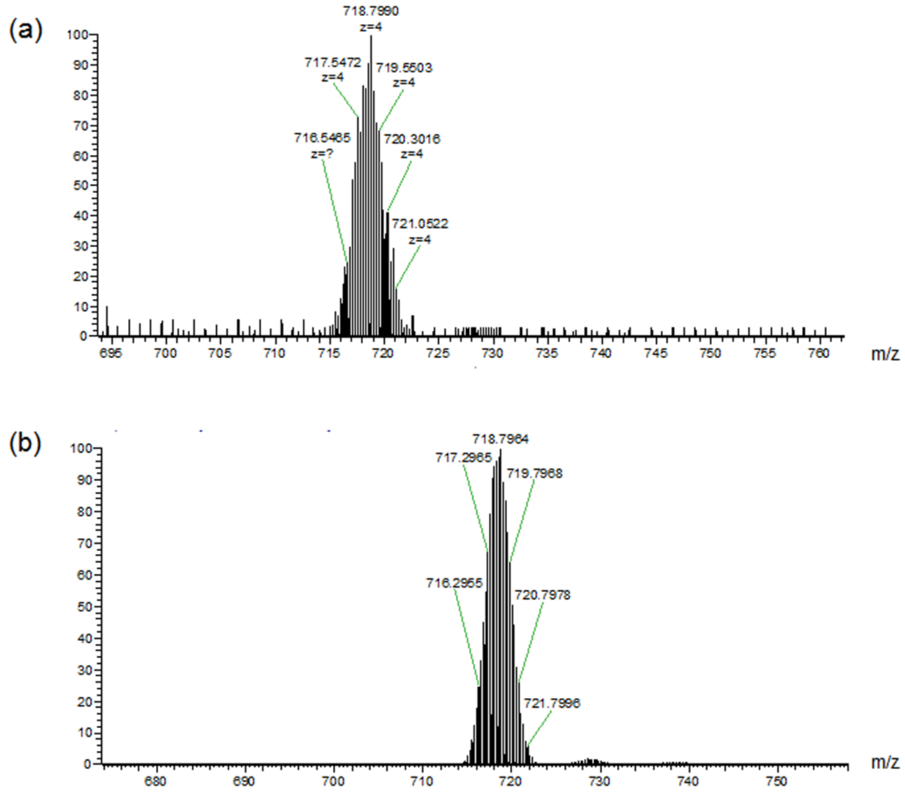

Figure S5. ESI-MS data of L1/SiW sample (a) and individual SiW sample in negatively ionic reflector mode.

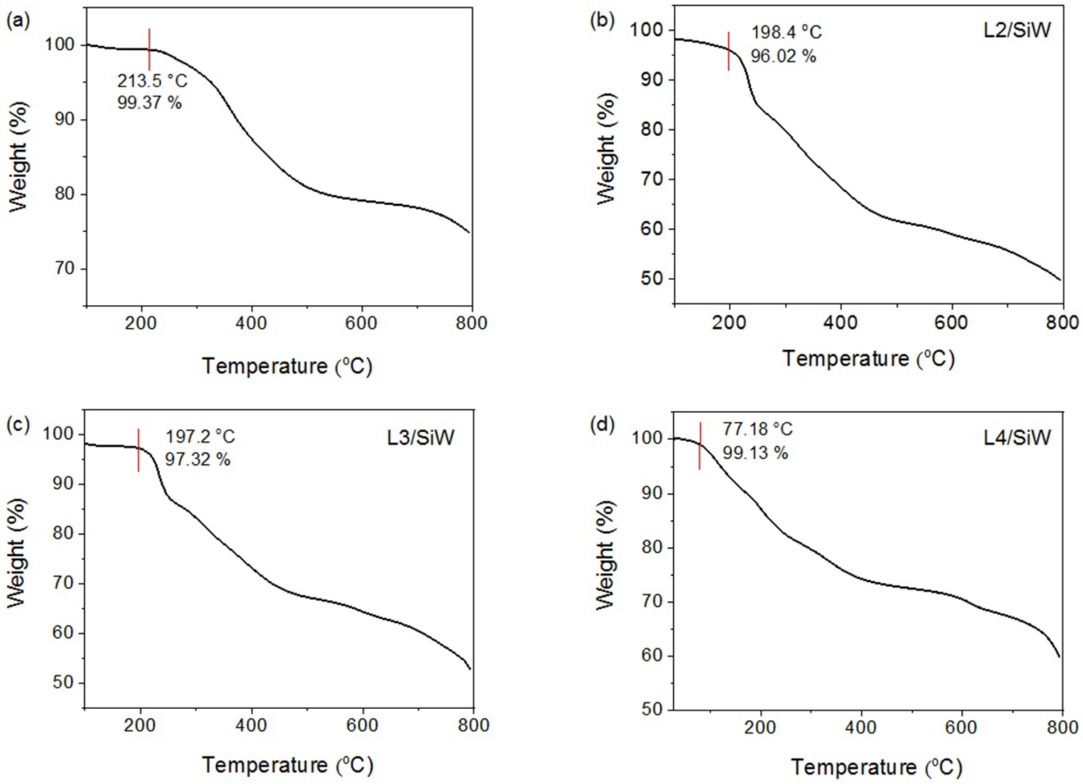

Figure S6. TGA curve of the lyophilized coacervate samples: (a) L1/SiW; (b) L2/SiW; (c) L3/SiW; (d) L4/SiW.

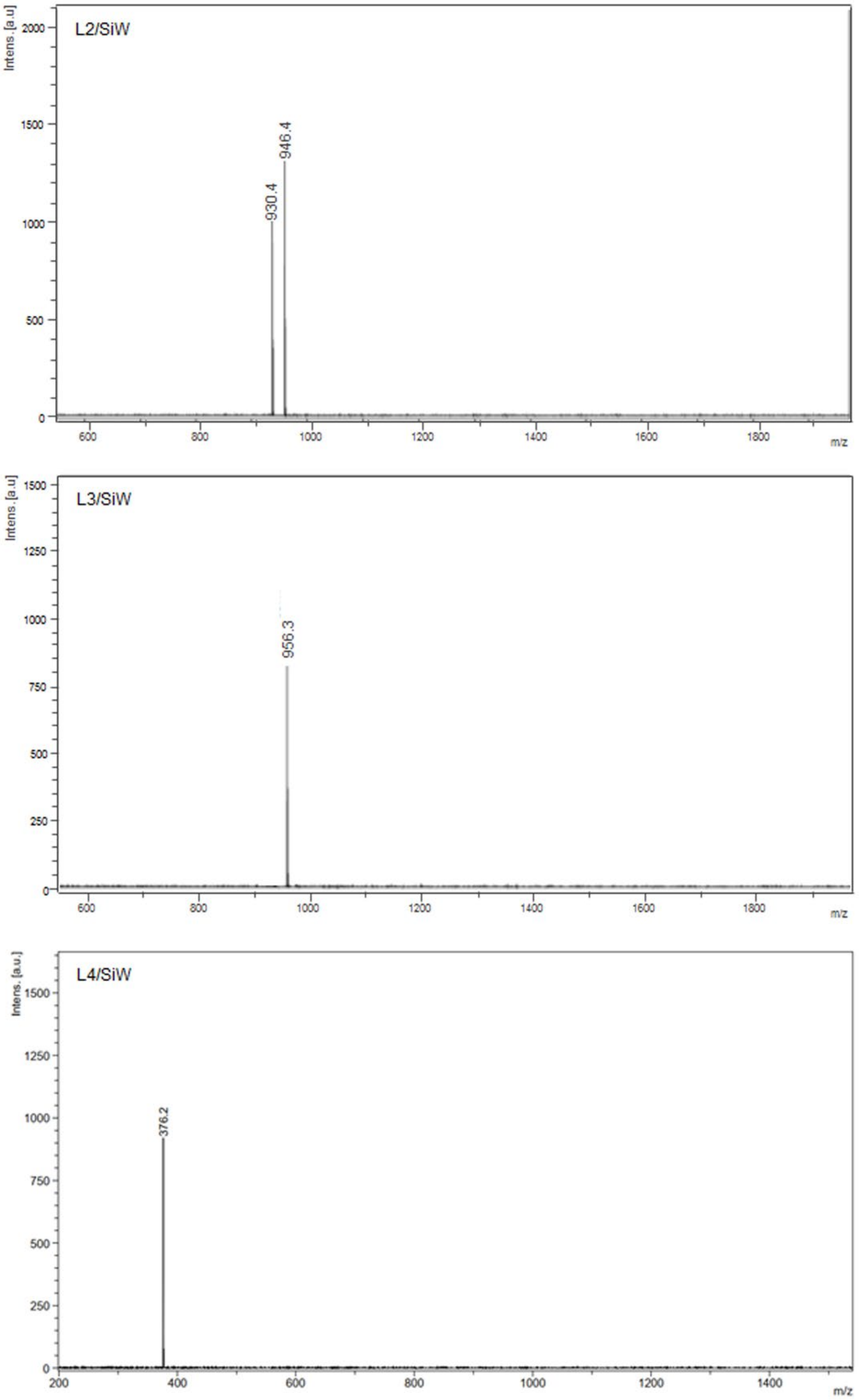

Figure S7. MALDI-TOF-MS data of diverse coacervate samples.

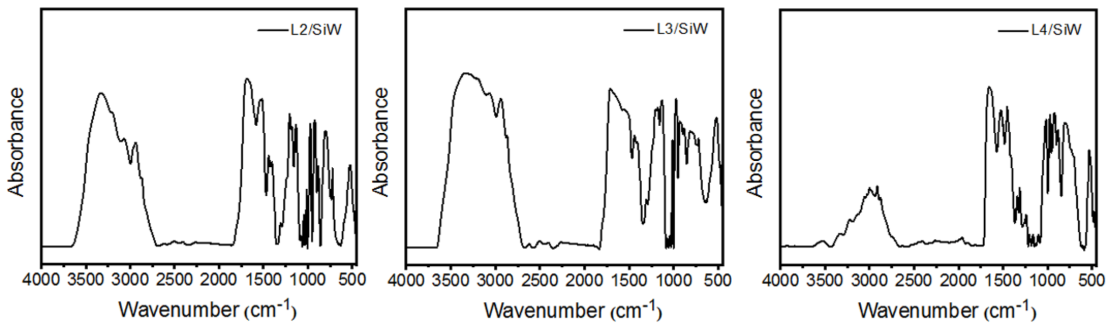

Figure S8. FT-IR spectra of L2/SiW, L3/SiW and L4/SiW coacervates.

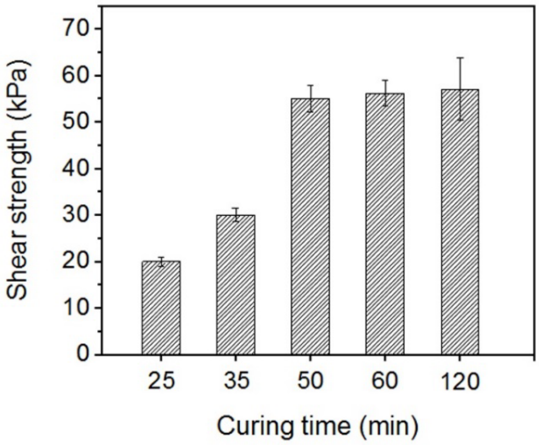

Figure S9. Shear adhesion strength of L1/SiW bonded on SS with different solvent-exchange time.

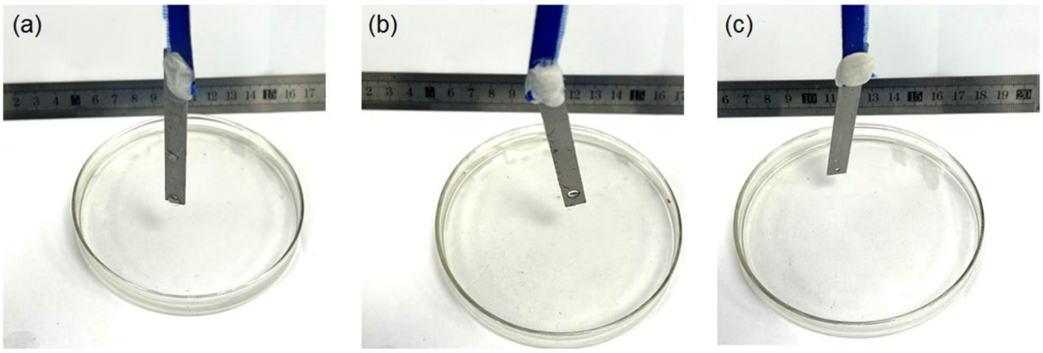

Figure S10. The solvent-exchange triggered curing of L4/SiW coacervate in tap water (a), river water (b) and 100 mM NaCl solution (c), respectively.
